# Dysfunctional interactions between the default mode network and the dorsal attention network in subtypes of amnestic mild cognitive impairment

**DOI:** 10.18632/aging.102380

**Published:** 2019-10-24

**Authors:** Junkai Wang, Jianghong Liu, Zhiqun Wang, Pei Sun, Kuncheng Li, Peipeng Liang

**Affiliations:** 1 School of Psychology, Capital Normal University, Beijing Key Laboratory of Learning and Cognition, Beijing, China; 2 Department of Psychology, Tsinghua University, Beijing, China; 3 Department of Neurology, Xuanwu Hospital, Capital Medical University, Beijing, China; 4 Department of Radiology, Aerospace Center Hospital, Beijing, China; 5 Department of Radiology, Xuanwu Hospital, Capital Medical University, Beijing Key Laboratory of Magnetic Resonance Imaging and Brain Informatics, Beijing, China

**Keywords:** Alzheimer's disease, amnestic mild cognitive impairment, resting state functional anticorrelations, default mode network, dorsal attention network

## Abstract

An anticorrelated relationship in the spontaneous fluctuations between the default mode network (DMN) and dorsal attention network (DAN) is a robust feature of intrinsic brain organization in healthy individuals. Prior studies have reported a decreased anticorrelation between the DMN and the DAN in Alzheimer's disease (AD) and mild cognitive impairment (MCI). However, it is unclear how this anticorrelation changes as MCI progresses to AD. We hypothesized that dysfunctional connectivity between the DMN and DAN may reflect the gradual decline from MCI to AD. To test this hypothesis, we investigated alterations in functional connectivity between the DMN and DAN in subtypes of amnestic MCI (aMCI) by comparing with the same functional pattern in healthy elderly individuals and patients with AD. We retrospectively collected brain imaging and neuropsychological data from 20 AD participants, 22 participants with multiple-domain aMCI (aMCI-m), 29 participants with single-domain aMCI (aMCI-s) and 23 sex-matched normal controls in this study. Resting-state functional connectivity analysis revealed that aMCI-s and aMCI-m groups demonstrated different magnitudes of increased anticorrelation between the DMN and DAN relative to the AD group. Furthermore, in aMCI-s, aMCI-m and AD participants, hypoconnectivity was found in specific regions within the DMN, including the precuneus and angular gyrus, and hyperconnectivity was found in areas outside the typical DMN networks, including the middle occipital gyrus, lingual gyrus and visual cortex, which indicated disease-related adaptations of brain networks. Our findings suggest that DMN-DAN anticorrelation may shed light on the understanding of the adaptations in brain function during the progression from MCI to AD and may serve as a potential biomarker to detect AD in the preclinical stage.

## INTRODUCTION

The early detection of Alzheimer's disease (AD) is key for postponing the development of the disease and has led people to focus on the earliest stage of the development of pathological processes, known as amnestic mild cognitive impairment (aMCI), which represents a probable transitional state between normal aging and early dementia [1, 2]. It has been reported that approximately 4% to 6% of people 65 years old or older have aMCI, which develops into AD at a rate of 10% to 15% yearly [3, 4]. Based on the pattern of cognitive deficits, aMCI can be further classified into single-domain aMCI (aMCI-s), characterized by isolated memory impairment, and multiple-domain aMCI (aMCI-m), characterized by multiple cognitive domain deficits, including memory and other cognitive functions [5]. Given that memory decline is usually the core and earliest symptoms of AD, aMCI-s may represent a very early stage of AD. As AD progresses, gradual decline of cognitive function spreads to other domains, resulting in aMCI-m [6]. According to this view, we speculate that aMCI-s is an earlier stage of AD than aMCI-m. This hypothesis is supported by a prior study and other studies which prove a higher risk of developing into AD in aMCI-m than in aMCI-s [6–8]. Thus, it is necessary to understand neurodegenerative changes that occur in aMCI-s and aMCI-m to identify possible biomarkers for detecting AD in its preclinical stage.

Previous evidence has consistently suggested that AD involves large-scale distributed networks and their complex interactions [9–12]. Resting-state functional magnetic resonance imaging (rs-fMRI), which has emerged as a noninvasive and systematic approach to explore large-scale networks and their interactions, is a fundamental tool offering a way to detect abnormal network alterations underlying neurodegenerative diseases such as AD [13–15]. Recent rs-fMRI studies have revealed that the human brain is intrinsically organized into functionally specialized regions that show a reciprocal pattern of spontaneous activity [16–18]. More specifically, two broad systems, the default mode network (DMN) and the dorsal attention network (DAN), oppose each other [16]. The DMN is mainly composed of the posterior cingulate cortex (PCC), medial prefrontal cortex (mPFC), bilateral angular gyrus (AG) and the hippocampus (Hp) and is engaged during internal processing [16, 19]. The DAN includes the intraparietal sulcus (IPS), frontal eye fields (FEF), superior parietal lobule (SPL) and the dorsolateral prefrontal cortex (dLPFC) and mediates external processing and attention-demanding cognitive functions [16, 20]. Anticorrelation between the DMN and DAN seems to reflect the brain’s network interactions and may serve as an essential neural substrate for flexibly allocating attentional resources, which is important for normal cognitive function [21, 22]. To be more specific, individuals with a stronger DMN-DAN anticorrelation during the resting state have been shown to perform better on cognitive tasks [23–25]. Even when performing the task, the DMN and the DAN showed opposing relationships, and a greater DMN-DAN anticorrelation was associated with higher cognitive control and better memory performance in normal subjects [26, 27]. Thus, increased anticorrelation between the DMN and DAN has been considered an index of efficient cognitive processing [26, 27].

During the various stages of life, functional interactions between the DMN and DAN are dynamically reciprocal. A previous study conducted during early infancy demonstrated that the anticorrelated behaviors between the DMN and DAN are absent at birth but have appeared by one year and are further enhanced during the second year of life [28]. From childhood to adulthood, the DMN-DAN anticorrelation becomes more robust and helps in the development of cognitive function [29, 30]. Transitioning into older adulthood, older adults showed a significantly attenuated anticorrelation between the DMN and DAN compared with that of young adults [31, 32]. Pathological changes that occur in AD may accelerate the aging process. Numerous studies have reported functional connectivity deficits within the DMN and DAN in AD and MCI participants [33–36]. Recent studies have also demonstrated that the anticorrelation between the DMN and DAN was decreased in MCI and AD participants. Moreover, dysconnectivity between the DMN and DAN might be potential predictors of AD progression [37–39]. However, it is still unclear how this anticorrelation changes across different stages of cognitive impairment, from normal aging to aMCI and, finally, AD.

In the current study, we aimed to characterize the alterations in functional connectivity between the DMN and DAN in subtypes of aMCI by comparing these patterns with the same functional patterns in healthy elderly individuals and patients with AD. Given that aMCI-s is an earlier stage of AD than aMCI-m, it is hypothesized that dysfunctional connectivity between the DMN and DAN may reveal a trend of gradual decline from MCI to AD. To test whether the dysfunction within the DMN and DAN may subsequently disturb functional anticorrelation between the DMN and DAN, we also examined altered functional connectivity within the DMN and DAN in subtypes of aMCI and AD.

## RESULTS

The images were visually inspected for excessive motion artifacts and distortions to ensure adequate quality. The head motion index was used to assess the motion of four groups. The average framewise displacement (FD: sum of the absolute values of the differentials of 3 translational and 3 rotational motion parameters) was calculated for each individual [40]. When comparing the average FD values across different groups using one-way ANOVA, we found no group differences among the four groups (F_3, 82_ = 0.949, P = 0.421).

Demographic data and behavioral assessments are listed in Table 1. Participants in AD, aMCI-m, aMCI-s and NC groups were well matched for sex, but there was a slight difference in age between the groups (F_3, 90_ = 3.47, P = 0.019). Post hoc analyses showed that individuals in the aMCI-s group were relatively older (P = 0.034) than NCs. Education level (F_3, 90_ = 3.47, P = 0.002) was significantly lower in the AD (P = 0.006) and aMCI-s (P = 0.015) groups than in the NC group. There were significant differences across the four groups in all the cognitive domains. Post hoc analyses revealed a significant trend of gradual impairment in all cognitive measures in all groups from NC to AD. Specifically, the AD group had the worst performance on all behavioral assessments relative to the other three groups. The MCI-m group also showed significant impairment in all cognitive domains compared with the NC group. In addition, the MCI-m group showed significantly impaired cognitive abilities compared with the MCI-s group, reflected in BNT, TMT and CDT scores. Moreover, the MCI-s group exhibited impaired performance in AVLT, MMSE and MoCA assessments compared with the NC group.

**Table 1:** Demographic and neuropsychological assessments of participants.

	**AD (n = 20)**	**aMCI-m (n = 22)**	**aMCI-s (n = 29)**	**Normal controls (n = 23)**	***P* Value**
Age, mean (SD), y	70.84 (9.51)	71.09 (8.41)	71.21 (6.48)	64.61 (9.32) **^c^**	0.019^*^
Sex, males/females	9/11	12/10	15/14	10/13	0.81^**#**^
Education, mean (SD), y	7.47 (4.02) ^**a**^	10.32 (3.72)	8.14 (3.89) ^**a**^	11.43 (3.38)	0.002^*^
AVLT scores, mean (SD)	12.68 (7.52) ^**abc**^	29.50 (11.12) ^**a**^	27.31 (5.87) ^**a**^	48.96 (9.07)	< 0.001^*^
BNT scores, mean (SD)	12.63 (6.78) ^**abc**^	23.36 (2.15) ^**ac**^	27.79 (1.35)	29.26 (0.86)	< 0.001^*^
TMT scores, mean (SD)	259.79 (48.37) ^**abc**^	114.09 (29.88) ^**ac**^	79.52 (23.35)	71.04 (36.52)	< 0.001^*^
MMSE scores, mean (SD)	16.58 (7.49) ^**abc**^	24.45 (4.04) ^**a**^	24.07 (3.47) ^**a**^	28.61 (1.50)	< 0.001^*^
MoCa scores, mean (SD)	12.11 (5.72) ^**abc**^	20.36 (4.47) ^**a**^	19.45 (4.24) ^**a**^	26.65 (1.92)	< 0.001^*^
CDT, (0, 1, 2, 3)	0 = 6, 1 = 7, 2 = 7 ^**abc**^	1 = 6, 2 = 9, 3= 7 ^**ac**^	2 = 5, 3 = 24	2 = 2, 3 = 21	**< 0.001^#^**
CDR, (0, 0.5, 1-2)	0.5 = 1, 1-2 = 19 ^**abc**^	0.5 = 22 ^**a**^	0.5 = 29 ^**a**^	0 = 23	**< 0.001^#^**

Note: One-way ANOVA and Chi-square analyses were applied to test for group differences, statistical significance level was set at *P* < 0.05 (two-tailed). Significant *P*-values were in bold. Superscript letters indicated whether group mean was significantly worse than healthy controls (a), aMCI-m group (b) and aMCI-s group (c), based on post hoc pairwise comparisons (Bonferroni corrected *P* < 0.05). ^*^ The p value was obtained using One-way ANOVA. ^#^ The p value was obtained using Chi-square test with multiple comparison. Abbreviations: AVLT: Auditory Verbal Learning Test; BNT: Boston Naming Test; TMT: Trail-Making Test; MMSE: Mini-Mental State Examination; MoCa: Montreal Cognitive Assessment; CDT: Clock-Drawing Test; CDR: Clinical Dementia Rating; AD: Alzheimer's disease; aMCI-m: Multiple-Domain Amnestic Mild Cognitive Impairment; aMCI-s: Single-Domain Amnestic Mild Cognitive Impairment.

**Figure 1:**
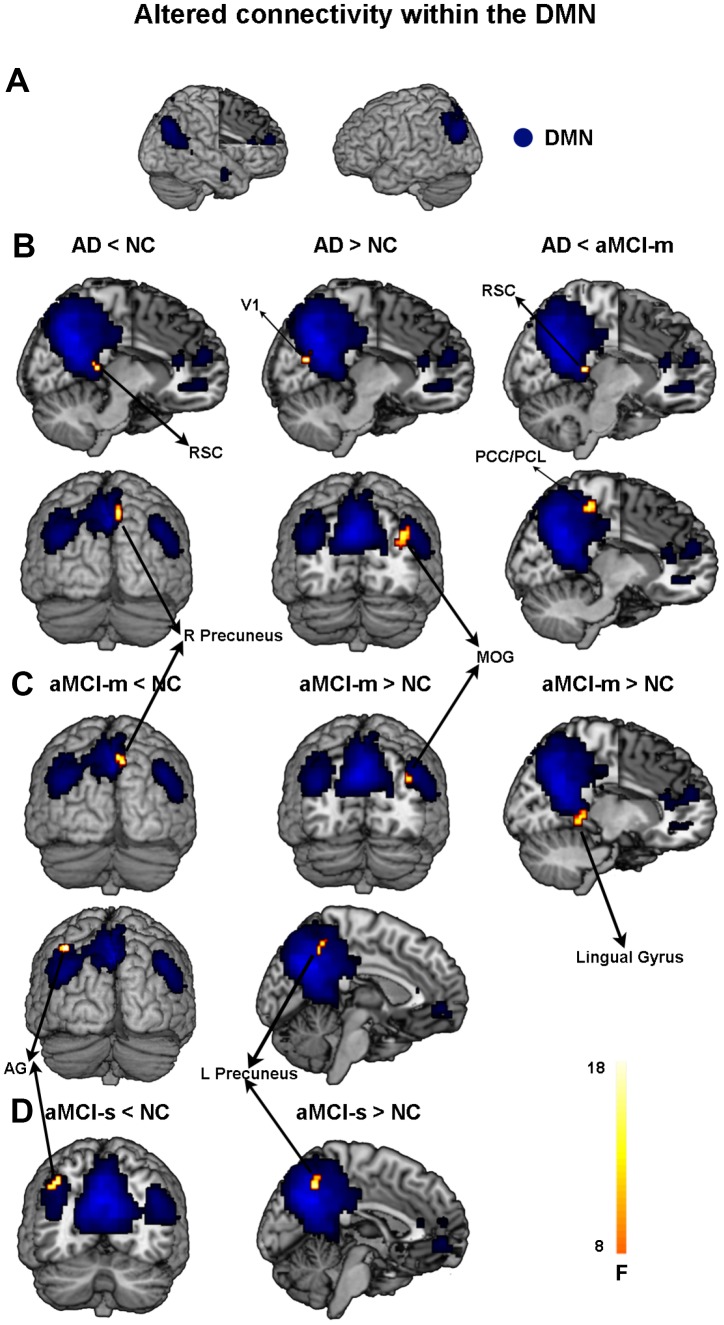
**Altered functional connectivity within the DMN among the four groups.** (**A**) Spatial distribution of the DMN (FWE correction). (**B**) Regions showing hypo- and hyperconnectivity within the DMN in the AD group; (**C**) Regions showing hypo- and hyperconnectivity within the DMN in the aMCI-m group; (**D**) Regions showing hypo- and hyperconnectivity within the DMN in the aMCI-s group. Abbreviations: DMN: default mode network; RSC: retrosplenial cortex; V1: primary visual cortex; MOG: middle occipital gyrus; PCC: posterior cingulate cortex; PCL: paracentral lobule; AG: angular gyrus; AD: Alzheimer's disease; aMCI-s: single-domain of amnestic mild cognitive impairment; aMCI-m: multiple-domain of amnestic mild cognitive impairment; NC: normal controls.

**Figure 2:**
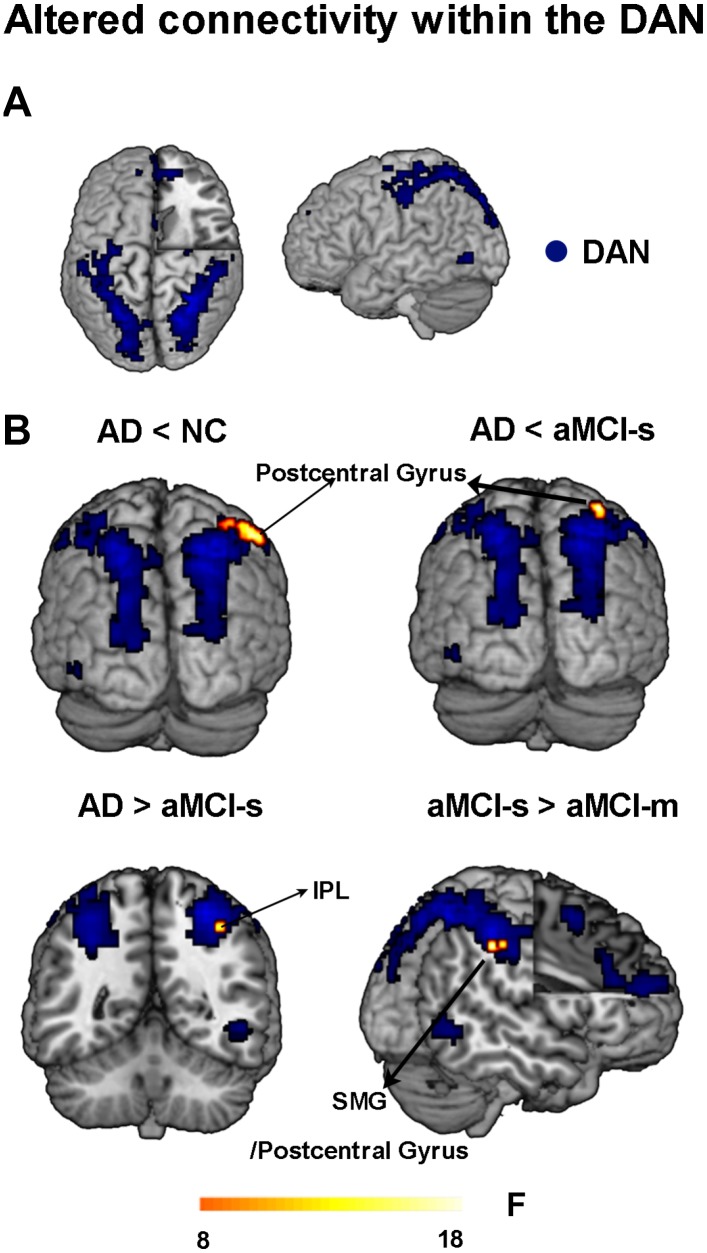
**Altered functional connectivity within the DAN among the four groups.** (**A**) Spatial distribution of the DAN (FWE correction). (**B**) Regions showing altered functional connectivity between different groups. Abbreviations: DAN: dorsal attention network; IPL: inferior parietal lobule; SMG: supramarginal gyrus; AD: Alzheimer's disease; aMCI-s: single-domain of amnestic mild cognitive impairment; aMCI-m: multiple-domain of amnestic mild cognitive impairment; NC: normal controls.

First, we found hypoconnectivity within the DMN and DAN in subtypes of aMCI and AD compared to that in NC, which is consistent with previous findings [11, 35, 36]. More precisely, the spatial distribution of the DMN and DAN was obtained across subtypes of aMCI, NC and AD groups (p < 0.05, FWE corrected; Figure 1A and 2A). For the DMN, the AD group had significantly decreased positive functional connectivity in the right precuneus and right retrosplenial cortex compared to NC (Figure 1B and Supplementary Table 2). There was also significantly decreased positive functional connectivity in the left angular gyrus and right precuneus in the aMCI-m group compared to NC (Figure 1C and Supplementary Table 2). The aMCI-s group showed significantly reduced positive functional connectivity in the left angular gyrus compared to NC (Figure 1D and Supplementary Table 2). For the DAN, only the AD group showed significantly reduced positive functional connectivity in the right postcentral gyrus compared to NC (Figure 2B and Supplementary Table 2). In addition, for the DMN, the AD group also showed significantly reduced positive functional connectivity in the right retrosplenial cortex and the right PCC extending to the right paracentral lobule compared to the aMCI-m group (Figure 1B and Supplementary Table 2). For the DAN, the AD group showed significantly decreased positive functional connectivity in the right postcentral gyrus compared to the aMCI-s group (Figure 2B and Supplementary Table 2). The aMCI-m group also showed significantly decreased positive functional connectivity in the right supramarginal gyrus extending to the right postcentral gyrus compared to the aMCI-s group (Figure 2B and Supplementary Table 2).

Besides hypoconnectivity, we also observed hyperconnectivity within the DMN in subtypes of aMCI and AD groups relative to NC. Compared with NC, AD showed significantly increased positive functional connectivity than NC in the right middle occipital gyrus and right primary visual cortex (Figure 1B and Supplementary Table 2). The aMCI-m group showed significantly increased positive functional connectivity in the left precuneus, right middle occipital gyrus and right lingual gyrus compared to NC (Figure 1C and Supplementary Table 2). The aMCI-s group only showed significantly increased positive functional connectivity in the left precuneus (Figure 1D and Supplementary Table 2).

**Figure 3:**
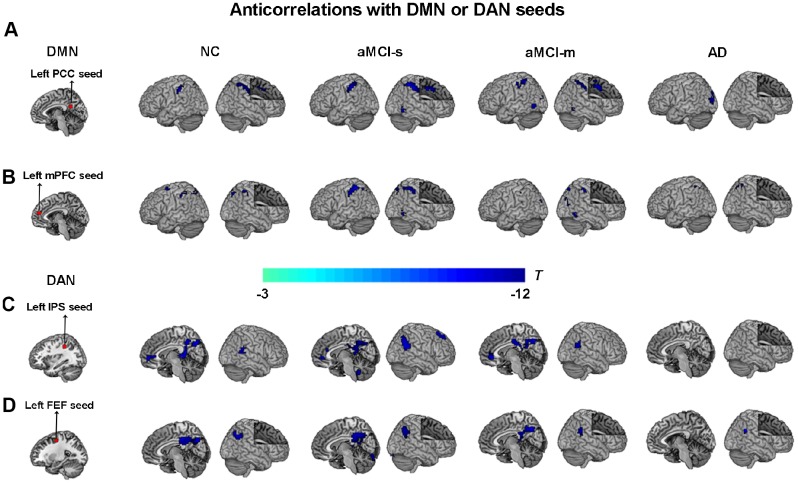
**Anticorrelations between the DMN and DAN among the four groups.** (**A**) The left PCC seed (red color) showing anticorrelations with the DAN (cool colors) among the four groups; (**B**) The left mPFC seed (red color) showing anticorrelations with the DAN (cool colors) among the four groups; (**C**) The left IPS seed (red color) showing anticorrelations with the DMN (cool colors) among the four groups; (**D**) The left FEF seed (red color) showing anticorrelations with the DMN (cool colors) among the four groups. P < 0.05, cluster level FWE corrected. Abbreviations: DMN: default mode network; DAN: dorsal attention network; PCC: posterior cingulate cortex; IPS: intraparietal sulcus; FEF: frontal eye fields; mPFC: medial prefrontal cortex; AD: Alzheimer's disease; aMCI-s: single-domain of amnestic mild cognitive impairment; aMCI-m: multiple-domain of amnestic mild cognitive impairment; NC: normal controls.

Second, to characterize the anticorrelations between the DMN and DAN in the aMCI subgroups, correlation maps of the DMN and DAN were employed. ROI-based intrinsic functional connectivity analysis revealed a similar anticorrelation pattern between the DMN and DAN for aMCI, NC and AD groups. The PCC and mPFC seeds showed strong negative correlations with the DAN, including the IPS, FEF, SPL, dlPFC, supplementary motor area and supramarginal gyrus extending to the postcentral gyrus (p < 0.05, cluster level FWE-corrected; Figure 3A and 3B). The IPS and FEF seeds showed strong negative correlations with the DMN, including the PCC, precuneus, mPFC, AG and superior frontal gyrus (p < 0.05, cluster level FWE-corrected; Figure 3C and 3D). As shown in Figure 3, relative to NC, anticorrelations between the DMN and DAN in aMCI-s and aMCI-m were enhanced at different magnitudes and were more expanded, but robustly attenuated in AD.

**Figure 4:**
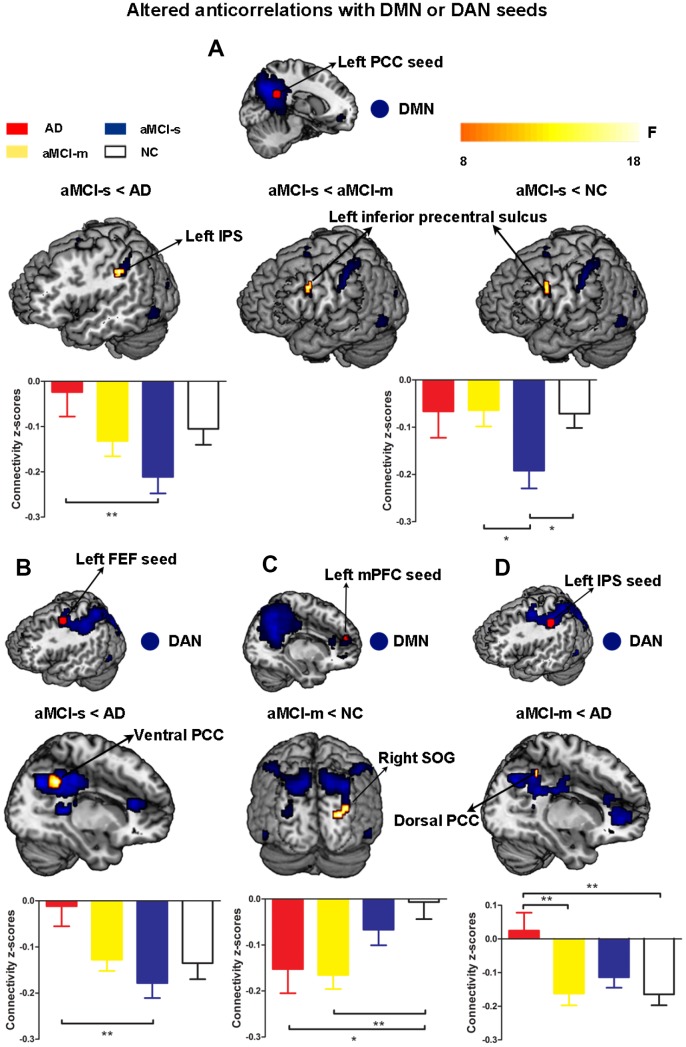
**Altered anticorrelations between the DMN and DAN among the four groups.** (**A**) Regions showing altered anticorrelations with the left PCC seed among the four groups; (**B**) Regions showing altered anticorrelations with the left FEF seed between the aMCI-s and AD groups; (**C**) Regions showing altered anticorrelations with the left mPFC seed between the aMCI-m and NC groups; (**D**) Regions showing altered anticorrelations with the left IPS seed between the aMCI-m and AD groups. Significant regions (shown in warm yellows) were overlaid on the main effect maps (shown in blue) which were corrected using a voxel threshold of FWE correction (*P* < 0.05). Bar graphs displayed mean rsFC z scores for the AD, aMCI-s, aMCI-m and NC groups and the error bars represented standard deviation. Mean rsFC z scores were analyzed with SPSS 20 using one-way ANOVA. *p < 0.05. **p < 0.01. Abbreviations: DMN: default mode network; DAN: dorsal attention network; PCC: posterior cingulate cortex; IPS: intraparietal sulcus; FEF: frontal eye fields; mPFC: medial prefrontal cortex; SOG: superior occipital gyrus; AD: Alzheimer's disease; aMCI-s: single-domain of amnestic mild cognitive impairment; aMCI-m: multiple-domain of amnestic mild cognitive impairment; NC: normal controls.

Third, to further examine dysfunctional connectivity between the DMN and DAN during the progression from MCI to AD, we directly compared each of the patient groups with the NC group. For the aMCI-s group, the results revealed significantly higher anticorrelation strengths between the left PCC seed and the left inferior precentral sulcus than in the NC and aMCI-m groups, as well as between the left PCC seed and the left IPS than in the AD group (Figure 4A and Supplementary Table 1). The aMCI-s group also showed the most significantly increased negative functional connectivity between the left FEF seed and the right ventral PCC compared to the AD group (Figure 4B and Supplementary Table 1). Moreover, the aMCI-m group had significantly increased negative functional connectivity between the left mPFC seed and the superior occipital gyrus compared to NC, as well as between the left IPS seed and the right dorsal PCC compared to the AD group (Figure 4C and 4D and Supplementary Table 1).

**Figure 5:**
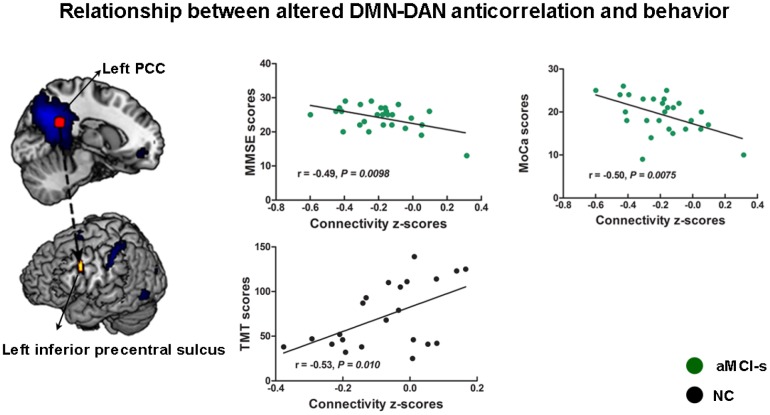
**The relationship between cognitive performance and the altered anticorrelations between the DMN and DAN across the aMCI-s and NC groups.** The connectivity strengths between the left PCC and left inferior precentral sulcus had correlations with the TMT scores, the MMSE scores and the MoCa scores. Brain maps of representative slices of related areas are also showed in the figure and colored dots represent their locations. Arrows are for illustrating purpose and do not imply directionality. Abbreviations: DMN: default mode network; DAN: dorsal attention network; PCC: posterior cingulate cortex; TMT: Trail-Making Test; MMSE: Mini-Mental State Examination; MoCa: Montreal Cognitive Assessment; aMCI-s: single-domain of amnestic mild cognitive impairment; NC: normal controls.

Finally, we examined the relationship between cognitive domains and the altered anticorrelations between the DMN and DAN among the four groups. The connectivity strengths between the left PCC and left IPS showed negative correlations with the BNT scores (r = -0.55, p = 0.028) and the MMSE scores (r = -0.49, p = 0.050) in the AD group and the AVLT scores (r = -0.38, p = 0.048) in the aMCI-s group (Supplementary Figure 1A). The connectivity strengths between the left PCC and left inferior precentral sulcus (IPrCS) had significant positive correlation with the TMT scores (r = 0.53, p = 0.010) in the NC group and significant negative correlations with the MMSE scores (r = -0.49, p = 0.0098) and the MoCa scores (r = -0.50, p = 0.0075) in the aMCI-s group (Figure 5). The connectivity strengths between the left IPS and the right dorsal PCC had negative correlation with the AVLT scores (r = -0.28, p = 0.050) in the aMCI-s and aMCI-m groups (Supplementary Figure 1B). To determine the significance, the Bonferroni correction was used to calculate the adjusted p-value corrected for multiple comparisons. The correlations between the left PCC-IPrCS connectivity strengths and cognitive scores in the NC or aMCI-s group were still significant after correction.

## DISCUSSION

The present study showed disease-related dysfunctional interactions during the progression from MCI to AD. Compared to the AD and NC groups, our results showed significantly increased anticorrelations between the DMN and DAN in the aMCI-s and aMCI-m groups, which revealed a coherent pattern of results with the behavioral analysis. Hyperconnectivity and hypoconnectivity were also observed within the DMN and DAN in the aMCI-s, aMCI-m and AD groups relative to NC, which revealed disease-related adaptations of brain networks and functional compensation. Furthermore, correlation analysis demonstrated that a stronger DMN-DAN anticorrelation during the resting state was associated with better cognitive performance among the four groups. These findings are critical for understanding adaptations in brain function during the progression from MCI to AD, and this DMN-DAN anticorrelation has the potential to detect AD in the preclinical stage.

### Altered anticorrelation strengths between the DMN and DAN

The anticorrelation between the DMN and DAN during rest is a core feature of the human brain's intrinsic architecture [16, 17]. This pattern remains stable when individuals perform cognitive tasks that demand various cognitive domains, including memory, cognitive control and visuospatial planning, and subjects with a stronger anticorrelation achieve better performance on cognitive measures [25, 27]. Our results of inter-network functional connectivity suggested that aMCI-s and aMCI-m groups showed different magnitudes of enhanced anticorrelation between the DMN and DAN relative to other groups. Based on the aforementioned information, these results were indicative of a neural mechanism of functional compensation that helps to improve cognitive deficits and maintain behavior at relatively normal levels.

Specifically, the aMCI-s group revealed increased anticorrelation strengths between the left PCC seed and the left IPS, as well as between the left FEF seed and the right ventral PCC, compared to the AD group. The aMCI-m group showed enhanced anticorrelation strengths between the left IPS seed and the right dorsal PCC compared to the AD group. These areas are central hubs of the DMN and the DAN. PCC sends dense projections to the medial temporal lobe, hippocampal formation, and parahippocampal cortex, which are important areas for memory [41]. Furthermore, it is particularly vulnerable to early deposition of amyloid beta-protein, one of the hallmark pathologies of AD [42]. IPS and FEF contribute to mediation of goal-directed process and orienting to external stimuli [20]. Prior studies demonstrated that attention deficits co-occurred with memory deficits in early stage AD [43, 44]. Consistent with the results of functional connectivity within the DMN and DAN, aMCI-s and aMCI-m groups used more functional resources of the DMN and DAN to compensate for cognitive function to perform tasks relatively normally. Our correlation analysis further demonstrated that better performance on various cognitive measures was associated with stronger negative functional connectivity between the DMN and DAN among the four groups. It is worth noting that both the dorsal and ventral PCC are important areas for memory [23, 43]. The results from a previous study showed that the ventral PCC had a closer relationship with ‘MCI/AD’ and can be seen as a more important central hub of the DMN [45]. This result may explain why the aMCI-s group showed more robust changes in functional connectivity in the ventral PCC, which could be utilized as a potential biomarker to detect early-stage AD.

Our present results also revealed that the aMCI-s group had a stronger anticorrelation between the left PCC seed and the left inferior precentral sulcus than NC and aMCI-m groups. Based on previous studies, the inferior frontal eye field and the inferior frontal junction area have been suggested to be located at the inferior precentral sulcus and are thought play critical roles in visual-biased attention, which may subsequently influence short-term memory [46, 47]. Considering the evidence mentioned above, enhanced anticorrelation strengths between the left PCC and the left inferior precentral sulcus in aMCI-s may rescue impaired memory. This result would also be useful to distinguish different subtypes of aMCI. Moreover, the aMCI-m group had significantly increased negative functional connectivity between the left mPFC seed and the superior occipital gyrus compared to NC. Our findings suggested that aMCI-m and AD groups showed hyperconnectivity in areas outside the typical networks. More specifically, we found that the aMCI-m group showed greater functional connectivity to the occipital gyrus within the DAN. Therefore, dysfunctional interactions between the DMN and DAN are not isolated and have a close relationship with functional changes within the DMN and DAN during the progression from MCI to AD. Hyperconnectivity in areas outside the typical DMN and DAN could subsequently disturb functional interactions between the DMN and DAN.

Overall, the aMCI-s group had the most significant increase in the DMN-DAN functional anticorrelation, and then, the increase in the anticorrelation for the aMCI-m group was lower in magnitude; comparatively, the anticorrelation strengths of the AD group were near-zero values. These findings suggested a gradient of DMN-DAN anticorrelation attenuation during the progression of the disease that was consistent with the behavioral assessments, revealing a significant trend of gradual impairment across different groups. A previous study also reported that the aMCI group showed an enhanced negative correlation between the salience network (SAL) and the DMN, which may compensate for and help sustain the anticorrelation between the DMN and DAN in early-stage AD [38]. Numerous studies have shown an attenuated anticorrelation between the DMN and DAN during the normal aging process both during task performance and at rest [31, 38, 39]. Reduced anticorrelation between the DMN and DAN in older adulthood is thought to be related to the poor modulation of attentional processes in response to shifting cognitive demands and inefficiency in processing cognitive resources [48–50]. In this way, the progression from MCI to AD may accelerate this process. In the early stage of pathological processes, different subtypes of aMCI patients still have the ability to allocate extra cognitive resources to compensate for different levels of cognitive deficits. With the progression of the disease, dysfunctional interactions between the DMN and DAN reach a significant level in AD patients, overcoming possible compensatory mechanisms, thus leading to gradual cognitive decline.

### Relationship between the DMN-DAN anticorrelation and functional connectivity within the DMN and DAN

Consistent with prior studies, our results replicated previous reports and showed reduced functional connectivity within the DMN and DAN in AD and MCI patients during the resting state [11, 35, 36]. Compared with NC, aMCI-m and AD groups showed reduced functional connectivity strength in the right precuneus within the DMN. The precuneus and the PCC comprise the core of the DMN and display the highest resting metabolic rates [51, 52]. These regions are involved in multiple cognitive processes, including, but not limited to, episodic memory, visuospatial processing, self-referential processing and consciousness [53]. We also observed that the AD group showed reduced functional connectivity in the right retrosplenial cortex within the DMN relative to NC. This area forms part of the PCC and has emerged as a crucial transit region between the hippocampus and cingulate cortex, having been implicated in a range of cognitive functions including episodic memory, spatial navigation and imagination [54–56]. Importantly, previous studies have indicated that atrophy of the retrosplenial cortex is present in the earliest clinical stage of AD and is a site often affected in AD [55, 56].

The aMCI-m and aMCI-s groups revealed decreased functional connectivity in the left angular gyrus compared to NC. A previous study demonstrated that the left angular gyrus is critical for episodic memory [57]. Our results showed that the aMCI-s group only had decreased functional connectivity in the left angular gyrus compared with NC, which is consistent with the definition of isolated memory impairment. Furthermore, we also found that the AD group showed reduced functional connectivity in the right retrosplenial cortex and the right PCC within the DMN compared to the aMCI-m group and had decreased functional connectivity in the right postcentral gyrus within the DAN compared to the aMCI-s group. The aMCI-m group showed decreased functional connectivity in the right supramarginal gyrus extending to the right postcentral gyrus compared to the aMCI-s group. Considering the aforementioned findings, our results supported a gradient of functional deficits. Observed hypoconnectivity in the aMCI-s, aMCI-m and AD groups relative to NC may reflect different levels of impairment among a variety of cognitive domains during the progression from MCI to AD. Preserved functional connectivity within the DMN and DAN may represent internal potential to maintain a reciprocal pattern between the DMN and DAN.

Hyperconnectivity was also observed within the DMN. Our results showed that aMCI-s and aMCI-m groups had increased functional connectivity in the left precuneus relative to NC. These findings can be explained by the compensation theory, which suggests that individuals with aMCI-s and aMCI-m may still have the ability to invoke the resources of the DMN to compensate for declining functional integrity. We also found that aMCI-m and AD groups, compared to NC, showed expanded connectivity to the middle occipital gyrus, which is outside the typical default network. In accordance with a previous study, these findings supported the concept of network expansion as a neural mechanism of functional compensation [58]. Additionally, aMCI-m and AD groups showed stronger and more extended connectivity to the lingual gyrus and the right visual cortex, respectively, than NC. These results demonstrated that individuals with aMCI-m and AD may need to recruit more cognitive resources from outside the typical default network to compensate for the declining integrity of the DMN. The altered functional connectivity within the DMN and DAN, including the hypoconnectivity in restricted regions in both networks and hyperconnectivity in areas outside the typical networks, may subsequently disturb functional interactions between the DMN and DAN.

Some limitations of this study need to be further discussed. First, age and educational level were not well matched across the four groups. However, the average age of all participants approached 65 years old. There were no significant differences among the aMCI-s, aMCI-m and AD groups and only a slight difference in age between the aMCI-s and NC groups. Therefore, age likely had little influence on our results. We also included educational level and age as covariates to avoid possible confounding effects on any observed differences in the functional connectivity of the brain among groups in our analysis. Second, although the sample size in the current study was relatively small, our results replicated the results from previous reports, and strict correlation maps between the DMN and DAN were employed to examine differences between aMCI, NC and AD groups. Future studies should increase the sample size to reveal more stable and objective differences between groups. Finally, although the present study revealed a gradient of deficits increasing from the NC group to the AD group, more cross-validation results and longitudinal studies are needed to better understand the progression of AD pathology.

## CONCLUSIONS

Overall, the present study provides evidence that hypoconnectivity in specific regions of both the DMN and DAN and hyperconnectivity in areas outside the typical networks were observed in aMCI-s, aMCI-m and AD groups, which may subsequently disturb functional interactions between the DMN and DAN. Furthermore, aMCI-s and aMCI-m groups showed different magnitudes of enhanced anticorrelation between the DMN and DAN, and then, the anticorrelation strength in the AD group dropped to nearly zero. This phenomenon suggested a gradient of DMN-DAN anticorrelation attenuation during the progression from MCI to AD. Finally, the dysfunctional anticorrelation between the DMN and DAN in subtypes of aMCI may have a large impact on behavioral performance. These results are important for understanding the adaptations in brain function during the progression of AD, and DMN-DAN anticorrelation may serve as a potential biomarker to detect AD in the preclinical stage.

## MATERIALS AND METHODS

### Participants

Our study sample included 20 AD participants (11 females), 22 aMCI-m participants (10 females), 29 aMCI-s participants (14 females) and 23 normal controls (NCs) (13 females). All AD and aMCI participants were recruited from the Department of Neurology at Xuanwu Hospital, Capital Medical University. The sex-matched NCs were recruited from the local community by advertisements. All participants received financial compensation for their participation. The experimental procedure was approved by the Research Ethics Committee of Xuanwu Hospital. Written informed consent was obtained from all participants or their relatives after the study had been fully explained.

All AD and aMCI participants underwent a detailed physical and neurological examination. They were screened by two experienced neurologists according to the standard criteria [59, 60]. The AD participants were assessed based on the Diagnostic and Statistical Manual of Mental Disorders-V (DSM-V) criteria for Alzheimer's Dementia, and the National Institute of Neurological and Communicative Disorders and Stroke/Alzheimer’s Disease and Related Disorders Association (NINCDS-ADRDA) criteria for AD [61–63]. Participants with aMCI were diagnosed and classified according to Petersen's clinical diagnostic criteria [59] and the National Institute on Aging-Alzheimer's Association criteria for MCI due to AD [60]. The inclusion criteria for aMCI were as follows: 1) memory complaint, preferably corroborated by an informant; 2) memory impairment for age; 3) essentially normal performance on general cognition and preserved activities of daily living; 4) Clinical Dementia Rating (CDR) score of 0.5 [64]; and 5) the absence of dementia. Exclusion criteria for all participants included the following: 1) cognitive impairment caused by head trauma or cranial surgery; 2) current or lifetime history of neurological or psychiatric disorders that could cause cognitive impairment, such as stroke, depression, or epilepsy; 3) neurological deficiencies, such as visual or hearing loss; and 4) contraindications to MRI. Demographic and behavioral data were analyzed with SPSS 20 using one-way ANOVA and chi-square tests. All demographic and neuropsychological assessment results are shown in Table 1.

### Neuropsychological assessment

All participants underwent an extensive battery of neuropsychological assessments that included the CDR [64], the Mini-Mental State Examination (MMSE) [65] and the Montreal cognitive assessment (MoCA) [66] as screening measures. Four cognitive domains were assessed: 1) memory function was measured with the Auditory Verbal Learning Test (short delay free recall) [67]; 2) naming skill was evaluated with the Boston Naming Test (BNT) [68]; 3) executive function was measured with the Trail Making Test (TMT) [69]; and 4) visuospatial ability was assessed with the clock drawing test (CDT; 3-point) [70]. The neuropsychological data were collected on the day before the MRI session.

### MRI data acquisition

Imaging data were acquired using a SIEMENS 3-Tesla scanner (Siemens Medical Solutions, Erlangen, Germany). Participants were instructed to keep their head still and their eyes closed and to refrain from thinking about anything in particular. Foam padding and headphones were used to limit head movement and minimize scanner noise. The high-resolution T1-weighted anatomical images were acquired using a multiecho magnetization prepared rapid gradient echo (MPRAGE) sequence. The acquisition parameters were as follows: repetition time = 1900 ms, echo time = 2.2 ms, inversion time = 900 ms, flip angle = 9°, acquisition matrix = 224 × 256 × 176, voxel size = 1 × 1 × 1 mm^3^. BOLD functional imaging was obtained in an axial orientation using a T2*-weighted, multislice gradient echo planar imaging (EPI) sequence with the following parameters: repetition time = 2000 ms, echo time = 40 ms, flip angle = 90°, acquisition matrix = 64 × 64, field of view = 256 mm × 256 mm, slice thickness = 4 mm, gap = 1 mm, 239 volumes, 28 slices, voxel size = 3.75 × 3.75 × 4 mm^3^.

### Image preprocessing

Preprocessing and analysis of rs-fMRI images were conducted with Statistical Parametric Mapping (SPM 8, University College London, London, UK; http://www.fil.ion.ucl.ac.uk/spm/software/spm8) and the CONN-fMRI functional connectivity toolbox v18a (http://www.nitrc.org/projects/conn [71]). Prior to preprocessing, the first 10 volumes of the functional images were discarded to allow for signal stabilization and the participants’ adaptation to the noisy environment. The remaining volumes were slice-time corrected (to the middle slice) and realigned to the first volume to reduce the confounding effects of head motion (using a 6 parameter rigid body transformation). Eight participants whose head motion exceeded 2 mm in translation or 2° in rotation were excluded from further analysis (4 AD participants, 1 aMCI-m participant, 2 aMCI-s participants and 1 healthy participant were excluded for this reason). We also calculated motion parameters (estimated by a realignment algorithm) for each group, and the difference between groups was statistically analyzed. For spatial normalization to the MNI space, the individual T1-weighted structural image was coregistered to the mean functional image. The T1 image was bias-corrected and segmented into gray matter (GM), white matter (WM), and cerebrospinal fluid (CSF) using template (ICBM) tissue probability maps. Parameters obtained from this step were subsequently applied to the functional data (resampled to 3 mm isotropic voxels) during normalization to the MNI space. Then, the data were spatially smoothed by a Gaussian filter with a full width half maximum (FWHM) of 6 mm.

Using the CONN toolbox, spurious sources of noise, such as heart rate and respiration signals, were estimated and regressed out using the anatomical component base noise reduction strategy (aCompCor) [72]. The aCompCor method efficiently removes principal components from WM and CSF regions. Specifically, the anatomical image for each participant was segmented into white matter, gray matter, and CSF. The white matter and CSF masks were eroded by one voxel, which resulted in substantially smaller masks than in the original segmentations. Then, the eroded white matter and CSF masks were used as noise regions of interest to extract signals from the unsmoothed functional volumes to avoid the additional risk of contaminating white matter and CSF signals with gray matter signals. Global signal regression was not used because it can artificially introduce anticorrelations into the BOLD signal [73]. The motion parameters (3 translations and 3 rotations), along with their first-order and second-order temporal derivatives, were also regressed out. The BOLD time series were simultaneously filtered with the recommended bandpass filter (0.009–0.08 Hz) during this step [74], followed by removal of the linear trend.

### Functional connectivity analysis

After data preprocessing, we first performed group spatial independent component analysis (ICA) to decompose the data into functional networks using the CONN toolbox and then to identify networks of interest [75]. Data reduction was performed by principal component analysis (PCA) to decrease computational complexity. Then, the resulting volumes were temporally concatenated, and PCA was performed again. After data reduction, we performed ICA decomposition using the Infomax algorithm to identify 20 independent components (ICs), which were suggested to provide a reliable identification of the DMN and DAN [76, 77], estimated by minimum description length criteria (MDL) [78]. ICs and time courses were back-reconstructed for each participant. After visually inspecting all ICs, we also calculated the spatial correlation values between ICs and the templates from the CONN network’s default networks file to identify the DMN and DAN networks. The functional connectivity of the DMN and DAN networks was converted to Z-values using Fisher’s r-to-z transform before further correlation analyses were performed. Next, one-sample t-tests were separately performed on the correlation maps of the DMN and DAN to obtain statistical functional connectivity maps of each network. The significance level was set at a voxel level of P < 0.05, corrected for multiple comparisons using familywise error (FWE). The results were saved as the masks for subsequent inter-network analysis.

Seed-based resting state connectivity analysis was employed to explore DMN-DAN interactions. Two regions of interest (ROIs) in the DMN and 2 ROIs in the DAN that had been created by a previous study that parcellated the brain based on 1000 participants were defined for analysis [79]. The mean time series of the DMN ROIs were extracted from the posterior cingulate cortex (PCC; MNI coordinates: -7, -52, 26) and the medial prefrontal cortex (mPFC; MNI coordinates: -7, 49, 18) using spherical seeds with a 6 mm radius. The mean time series of the DAN ROIs were extracted from the intraparietal sulcus (IPS; MNI coordinates: -34, -38, 44) and the frontal eye field (FEF; MNI coordinates: -22, -8, 54) using spherical seeds with a 6 mm radius. Then, seed-based resting state connectivity maps were generated for each participant by calculating the correlation coefficients between the mean signal of each ROI and the time series at each voxel of the whole brain. Individual correlation maps were converted to Z-value maps using Fisher’s r-to-z transformation to improve the normality of the correlation coefficients. A one-sample t-test was performed separately for HC, AD, aMCI-m and aMCI-s participants on the correlation maps to identify the brain areas that were negatively correlated with each seed with a voxel threshold of P < 0.05, corrected for multiple comparisons using FWE. The resulting statistical maps for each group were subsequently combined within each ROI to generate specific masks for inter-network analysis. A voxelwise one-way ANOVA was performed to compare the intra-network functional connectivity difference between the DMN and DAN networks of the four groups. The inter-network functional connectivity represented the ROIs of the DMN or DAN connectivity maps, masked with correlation maps of the DAN or DMN, and the specific mask that negatively correlated with the corresponding DMN node or DAN node, as defined above.

### Statistical analyses

Statistical analysis was conducted for group differences using one-way analysis of variance (ANOVA) with group as a factor (4 groups: HC, aMCI-s, aMCI-m and AD) after regressing out the influence of age, education years and global correlation (GCOR). The main effect map of the group was first generated to create a sample-specific mask with a voxel threshold of the FWE correction (P < 0.05). Post hoc comparisons were used to identify meaningful intra-network and inter-network functional connectivity differences among the four groups within the corresponding sample-specific mask. The statistical significance was AlphaSim corrected at a voxel level of P < 0.05, which was obtained by clusters with a minimum size of 10 voxels and an individual voxel height threshold of P < .005, according to a Monte Carlo simulation (1000 simulations, FWHM = 4 mm) within the mask using REST software (http://restfmri.net/forum/REST_V1.8 [80]). To investigate the relationship between network interaction and cognitive ability, we also explored Pearson’s correlations between the behavioral data and the inter-network functional connectivity with significant between-group differences among aMCI-s, aMCI-m, AD and NC groups. A value of P < 0.05 was considered statistically significant.

## Supplementary Material

Supplementary Figure 1

Supplementary Tables
