# Supplementary material for: Dysfunctional interactions between the default mode network and the dorsal attention network in subtypes of amnestic mild cognitive impairment
Source: Aging (Albany NY). 2019 Oct 24;11(20):9147–66. doi: 10.18632/aging.102380 (PMC6834429; doi:10.18632/aging.102380)
Supplement: Supplementary Figure 1 [file aging-11-102380-s002.pdf]

SUPPLEMENTARY FIGURE

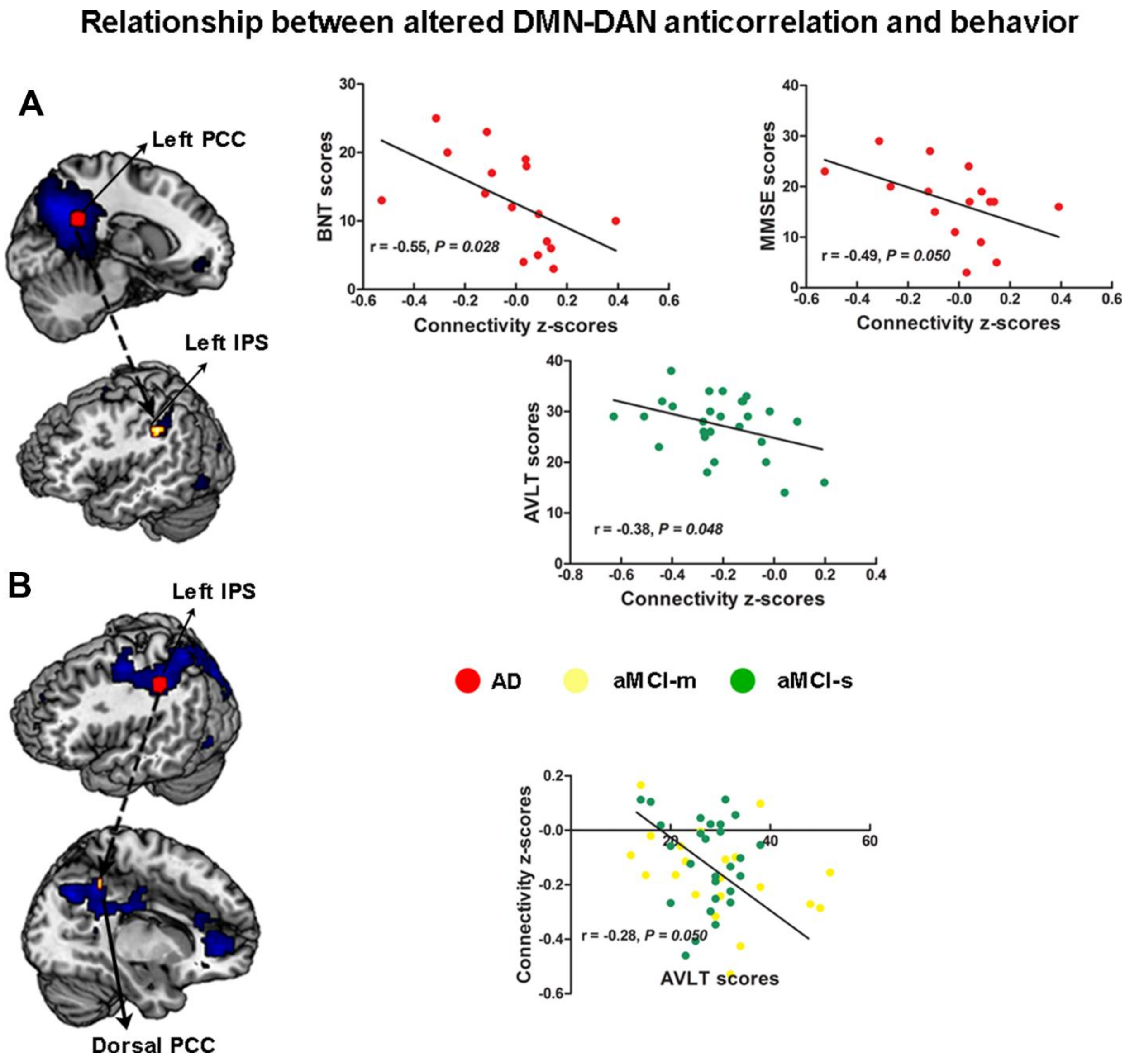

Supplementary Figure 1. The relationship between cognitive performance and the altered anticorrelations between the DMN and DAN across the aMCI-s, aMCI-m and AD groups. (**A**) The connectivity strengths between the left PCC and left IPS showed correlations with the AVLT scores, the BNT scores and the MMSE scores; (**B**) The connectivity strengths between the left IPS and the right dorsal PCC had correlations with the AVLT scores. (**A**–**B**) Brain maps of representative slices of related areas are also showed in the figure and colored dots represent their locations. Arrows are for illustrating purpose and do not imply directionality. Abbreviations: DMN, default mode network; DAN, dorsal attention network; PCC, posterior cingulate cortex; IPS, intraparietal sulcus; AVLT, Auditory Verbal Learning Test ; BNT, Boston Naming Test; MMSE, Mini-Mental State Examination; AD, Alzheimer's disease; aMCI-s, single-domain of amnestic mild cognitive impairment; aMCI-m, multiple-domain of amnestic mild cognitive impairment
