# Supplementary material for: Dysfunctional interactions between the default mode network and the dorsal attention network in subtypes of amnestic mild cognitive impairment
Source: Aging (Albany NY). 2019 Oct 24;11(20):9147–66. doi: 10.18632/aging.102380 (PMC6834429; doi:10.18632/aging.102380)
Supplement: Supplementary Tables [file aging-11-102380-s001.pdf]

SUPPLEMENTARY TABLES

Supplementary Table 1. Altered anticorrelations between the DMN and DAN among the four groups.

Seed	Brain regions	BA	side	Cluster size	F value	Peak MNI coordinates		
						X	Y	Z
**Left PCC**								
MCI-s < AD	IPS	40	L	10	11.09	-42	-36	38
MCI-s < MCI-m	IPrCS	6	L	12	13.07	-54	10	36
MCI-s < NC	IPrCS	6	L	22	17.76	-54	10	38
**Left mPFC**								
MCI-m < NC	SOG	19	R	12	9.82	34	-82	30
**Left FEF**								
MCI-s < AD	Ventral PCC	23	R	107	13.56	6	-52	36
**Left IPS**								
MCI-m < AD	Dorsal PCC	31	R	14	8.91	4	-48	42

Abbreviations: R: Right, L: left. MNI: Montreal Neurological Institute; BA: Brodmann areas; PCC: posterior cingulate cortex; IPS: intraparietal sulcus; FEF: frontal eye fields; mPFC: medial prefrontal cortex; IPrCS: inferior precentral sulcus; SOG: superior occipital gyrus; AD: Alzheimer's disease; aMCI-s: single-domain of amnestic mild cognitive impairment; aMCI-m: multiple-domain of amnestic mild cognitive impairment; NC: normal controls.

Supplementary Table 2. Altered functional connectivity within the DMN and DAN among the four groups.

Network	Brain regions	BA	Side	Cluster size	F value	Peak MNI coordinates		
						X	Y	Z
**DMN**								
AD < MCI-m	PCC/ RSC	30	R	16	13.47	10	-40	8
	PCC/PCL	31/5	R	30	11.84	6	-36	50
AD < NC	Precuneus	7	R	27	14.51	10	-76	54
	RSC	30	R	11	9.05	12	-42	12
AD > NC	MOG	19	R	58	12.63	40	-68	36
	V1	17	R	12	10.11	4	-66	16
MCI-m < NC	precuneus	7	R	72	12.45	6	-72	52
	Angular gyrus	39	L	19	10.18	-36	-70	50
MCI-m > NC	Lingual gyrus	19	R	21	16.53	12	-42	0
	Precuneus	7	L	23	11.41	-5	-54	44
	MOG	19	R	14	8.82	40	-70	32
MCI-s < NC	Angular gyrus	39	L	25	10.20	-42	-56	48
MCI-s >NC	Precuneus	7	L	46	11.18	-12	-54	48
**DAN**								
AD < MCI-s	Postcentral gyrus	1	R	22	13.52	34	-42	72
AD > MCI-s	IPL	40	R	27	13.00	36	-50	50
AD < NC	Postcentral gyrus	1	R	87	17.93	52	-30	56
	Postcentral gyrus	3	R		11.35	44	-32	60
	Postcentral gyrus	2	R	18	10.65	24	-46	60
MCI-m < MCI-s	SMG	40	R	26	10.52	48	-32	44
	Postcentral gyrus	3	R		8.83	42	-26	42

Abbreviations: R = Right, L = left. MNI: Montreal Neurological Institute; BA: Brodmann areas; DMN: default mode network; DAN: dorsal attention network; PCC: posterior cingulate cortex; RSC: retrosplenial cortex; PCL: paracentral lobule; V1: primary visual cortex; MOG: middle occipital gyrus; IPL: inferior parietal lobule; SMG: supramarginal gyrus; AD: Alzheimer's disease; aMCI-s: single-domain of amnestic mild cognitive impairment; aMCI-m: multiple-domain of amnestic mild cognitive impairment; NC: normal controls.
